# Repair of bone defects in rat radii with a composite of allogeneic adipose-derived stem cells and heterogeneous deproteinized bone

**DOI:** 10.1186/s13287-018-0817-1

**Published:** 2018-03-27

**Authors:** Jia Liu, Peng Zhou, Yu Long, Chunxia Huang, Danna Chen

**Affiliations:** 1 Department of Human Anatomy, Histology and Embryology, School of Basic Medical Sciences, Changsha Medical University, Changsha, 410219 China; 2 School of Biological Science and Technology, Central South University, Changsha, 410013 China

**Keywords:** Adipose-derived stem cells, Differentiation, Osteogenesis, Deproteinized bone, Bone defect, Molybdenum target X-ray

## Abstract

**Background:**

In the bone tissue engineering domain, seed cells, scaffold and cell-scaffold composites are three focuses. In this study, the feasibility of using allogeneic adipose-derived stem cells(ADSCs) combined with heterogeneous deproteinized bone (HDB) to repair segmental radial defects was investigated by observing the repair of the defect area.

**Methods:**

ADSCs were cultured in vitro, purified, antigen-detected and osteogenic differentiation potency-measured; then, the ADSCs of the third generation were seeded into HDB to prepare an ADSCs-HDB composite partly with osteogenesis induced cells. Sixty Wistar rats were randomly divided into four groups with 15 in each group. A bone defect (4 mm in length) was created at the left radius in each rat. Two kinds of ADSCs-HDB composites were implanted in the ADSCs osteogenesis group or ADSCs group; HDB was implanted in the negative control group; nothing was filled in the blank control group. The bone defect repair was evaluated by gross observation, molybdenum target X-ray examination and histological analyses after surgery.

**Results:**

Gross observation: the bone defect area was completely filled and difficult to recognize in the ADSCs osteogenesis group. The connection of the ADSCs group was strong, but the implants were clearly identifiable. The joints of the negative control group were slightly thick but the connection was unstable. In the blank control group, kermesinus tissue was between the two ends and bones were not connected after 8 weeks. Molybdenum target X-ray examinations: In the ADSCs osteogenesis group, evident bridges in the graft were observed in the defects in the fourth week; the defects were filled with new bone completely and a marrow cavity appeared at 8 weeks. In the ADSCs group, there were some callus formations, but the radial defect was still obvious at 8 weeks. In the negative control group, fracture lines were clear. In the blank control group, no osseous bridges were observed, which resulted in bone nonunion eventually in 8 weeks. There were significant differences in the callus density between experimental groups and the blank control group at 4 and 8 weeks (*P* < 0.01). Histological measures showed that the rate and quality of the new bone formation and remodelling was significantly different between the experimental and control groups.

**Conclusions:**

A composite of ADSCs-HDB has a strong osteogenic ability. It can repair segmental bone defects well and is promising to serve as grafting material in bone tissue engineering.

## Background

After trauma or tumour resection, large bone defects repair slowly and inefficiently if it is just natural repair mechanisms. In the 1990s, studies proposed the use of tissue engineering composite bone for complement, creating a new way of biological repair [1]. There are many kinds of seed cells that can be used in tissue engineering, in which mesenchymal stem cells (MSCs) show enhanced therapeutic properties [2]. MSCs could be obtained from different tissues, including bone marrow, peripheral blood, adipose tissue and umbilical cord. Recently, other new sources have been studied. The MSCs of adipose tissue, also known as adipose-derived stem cells (ADSCs), are the advantageous choice. There is not a single source. They can be easily collected from different parts of the human body and in high amounts. Because of ADSCs’ high differentiative capacities, they are capable of repairing vital tissues and organs, such as bone, cartilage, fat, liver, etc. And not only that, a systematic search in 70 studies involving more than 1400 patients who were treated with adipose-derived cell therapy showed a favourable safety profile [3]. Compared with stem cells from bone marrow, ADSCs exhibit enhanced proliferative capacity and retain multipotency longer during expansion in vitro [4]. They can be obtained by liposuction easily, which causes minimal harm to human beings, unlike bone marrow puncture, and they do not involve serious ethical issues [5]. For greater efficiency, the technical details for ADSCs repair of bone defects are not fully clear. It is widely believed that the success rate of the repair process depends on the interaction between ADSCs and the scaffold holding and supporting cells. The deproteinized bone has natural pores for cells. Furthermore, without the allogenic proteins stimulating immune responses, the heterogeneous deproteinized bone (HDB) has no serious immunogenicity. Therefore, allogeneic ADSCs combined with HDB were prepared and then implanted into a radial defect in rats in this study, so that a reasonable application of ADSCs’ osteogenesis ability in vivo could be studied.

## Methods

### Experimental design

Wistar rats (*N* = 76), 12-week-old males weighing 320–380 g, neonatal rats (*N* = 8) and 2-month-old male New Zealand rabbits (*N* = 4) were purchased from the experimental animal centre of the Third Xiangya Hospital of Central South University (Changsha, China) and housed in the standardized animal centre of Changsha Medical University with free access to food and water for 1 week prior to the experiment. The experiment consisted of two parts. In part one, 16 Wistar rats were used to separate ADSCs, eight neonatal rats to provide fibroblasts for comparison and few New Zealand rabbits to supply cancellous bone for production of HDB. In part two, 60 rats were randomly divided into four groups: the ADSCs osteogenesis group, the ADSCs group, the negative control group and the blank control group, in order to compare the effect of two composites and HDB alone on the defect repair.

### Separation, culture and authentication of ADSCs

The methods for acquiring ADSCs are mainly based on the previous studies with some modifications [6, 7]. Adipose tissue cells were isolated from Wistar rats. Briefly, the rats were euthanized by cervical vertebra dislocation under anaesthesia. Subcutaneous adipose tissue was carefully excised from the inguinal regions and washed with sterile phosphate-buffered saline (PBS) containing penicillin (100 u/ml) and streptomycin (100 μg/ml) to remove contaminating blood cells. The tissue was minced into pieces of 1 mm^3^ and digested in PBS with 0.05% collagenase type I (Sigma-Aldrich, St. Louis, MO, USA) for 60 min at 37 °C with constant vigorous agitation. The top lipid layer was removed, and the remaining portion was centrifuged at 200 g for 10 min at room temperature to separate the stromal cells from the floating adipocytes. The cells were then resuspended in Dulbecco’s modified Eagle’s medium (DMEM)/F12 medium (Gibco, Waltham, MA, USA) containing 10% newborn bovine serum (Invitrogen, Carlsbad, CA, USA) and the antibiotics described above. The cells were cultivated at 37 °C, 5% CO_2_ and 95% humidity. After 24 h, the unattached cells were removed by rinsing with PBS.

Cell surface antigen profiles were evaluated by flow cytometry. Flow cytometry analysis determined the percentage of specific markers in all the analysed cells. After reaching 80% cell confluence, adherent cells were removed by trypsinization (Invitrogen), washed twice with PBS and re-suspended at a density of 10^6^/ml. All cells were incubated with fluorescein-isothiocyanate-conjugated mouse anti-rat monoclonal antibodies against rat CD44, CD45, CD90 and CD105 (BD Biosciences, San Jose, CA, USA) at 4 °C for 40 min at a dilution of 1:100. Ten thousand stained cells were acquired and analysed by FACS Calibur flow cytometer using CellQuest 6.0 software (Becton Dickinson, Franklin Lanes, New Jersey, USA).

### Osteogenic induction and test of ADSCs

ADSCs were induced using DMEM/F12 supplemented with 1 nM dexamethasone, 2 mM β-glycerolphosphate and 50 μM ascorbate-2-phosphate for 7 days or 14 days in 24-well plates, and the osteogenic medium was changed every 2 days. Mineralization was assessed by alkaline phosphatase (ALP) enzymatic activity of ADSCs.

ADSCs were differentiated for 7 days or 14 days in osteogenic medium. ALP enzymatic activity of them was quantified by measuring the formation of p-nitrophenol from p-nitrophenyl phosphate, based on the unification cell number. After adding 1% Triton X-100 and repeatedly drying to drain the fluid from the cytoplasm, the experiments of the suspension were carried out according to the ALP test kit (Nanjing Jianchen Bioengineering, Nanjing, Jiangsu, China). The absorbance of every reaction system was measured at 520 nm. In addition, an equivalent number of fibroblasts acquired from neonatal rats was treated uniformly as the control group. ALP activity (king’s unit/100 ml) = (OD_measure_ - OD_blank)_÷(OD_standard_ - OD_blank_) × 0.1 mg/ml × 100 ml (1 king’s unit is defined as the production of 1 mg of phenol by 100 ml samples mixed with matrix for 15 min at 37 °C). The formula was used to calculate ALP activities for comparison.

### Preparation of HDB

The methods of preparing the HDB reference the related research [8]. The cancellous bones of the femora were collected from New Zealand rabbits; then, soft tissue, cartilage and bone marrow were removed. The bones were cut into cuboid-shaped sticks with dimensions of 4 mm × 1.5 mm × 1.5 mm. The bone sticks were digested in 0.3 mg/ml pepsin (Sigma-Aldrich) dissolved in pH 2.0 phosphate buffer (1.97 g NaH_2_PO_4_, 1.20 ml H_3_PO_4_ in 100 ml ddH_2_O) at 25 °C for 8 h. PH was adjusted to 9.0 with NaOH to deactivate the pepsin. After washing with ddH_2_O for 30 min, the HDBs were dried before use.

### ADSCs seeded onto HDB and adhesion rate calculation

ADSCs of the third generation were cultured in osteogenic medium for 14 days before adjusting the density to 10^6^ cells/ml by trypsinization. For the preparation of implant materials of the ADSCs osteogenesis group, 0.1 ml (10^5^ cells) was seeded carefully onto the HDB in a 96-well plate and incubated for 4 h. In addition, the same amount of ADSCs was inoculated onto the surface of the HDB for 4 h for the ADSCs group, which had not been osteogenesis induced. The ADSCs in or on HDB were digested by trypsin for 8 min in separate centrifuge tubes and rinsed with PBS twice. Then all the cells in trypsin and PBS were collected, trypan-blue stained and counted as adhesion number (AN). The AN of the control group was the number of cells adhering to a 96-well culture plate within 4 h. Adhesion rate was calculated though AN*10^− 5^*100%. The survival rates were determined by the proportion of trypan blue staining cells.

### Radial defect model preparation and composite implantation

Sixty Wistar rats were anaesthetized by 3% pentobarbital intravenously. Incisions were made from the lateral side of the left radius, and the muscles and subcutaneous tissues were blunt-dissected to dissociate the radius. A 4-mm-long bone defect was created surgically and the periosteum was removed near the two ends to prevent periosteum ossification. The rats were divided into four groups (*N* = 15 in each group), and then different graft materials were implanted, except for the blank control group. The HDBs were combined with the ADSCs, with or without osteogenesis differentiation, for the ADSCs osteogenesis group and the ADSCs group, and the HDBs alone were implanted for the negative control group; in the meantime, the defects were not filled with any material in the blank control group. Implants were put in the defect without being fixed, and then the muscle fascia was stitched, and the incision was closed. All surgical procedures were performed under sterile conditions. After surgery, the rats were given 20,000 U/d gentamycin by intramuscular injection for 3 days and housed separately.

### Molybdenum target X-ray examinations

Randomly selected five rats in each group were anaesthetized separately by batch at the time of 4 or 8 weeks post-operation. The posteroanterior radiographs of the rats were taken by an ultra-high sensitive molybdenum target X-ray apparatus. Image pro plus 6.0 Image analysis software (Media Cybernetics, Inc., Rockville, MD, USA) was used to selected the callus area to measure density values by the histogram of the callus parts.

### Gross and histology observation

Five rats in each group were sacrificed at 2, 4 and 8 weeks after implantation. The dissected radii were cleaned and immersed in a 10% formaldehyde solution, and then decalcification was carried out by immersing in 1 mol/L HCl for 12 h. The samples were subsequently dehydrated and embedded in paraffin. Haematoxylin and eosin (H&E) staining was performed and the samples were observed under light microscopy.

### Data analysis

Data were expressed as the means±SD. SPSS 10.0 software (SPSS Inc, Chicago, IL, USA) was used to analyse the data. Differences between the two groups were estimated by Student’s *t* test; differences among three or more groups were determined by a one-way ANOVA; comparisons between groups were carried out by the LSD *t* test. *P* < 0.05 was considered statistically significant.

## Results

### Morphological observation and antigen-detection of ADSCs

**Fig. 1:**
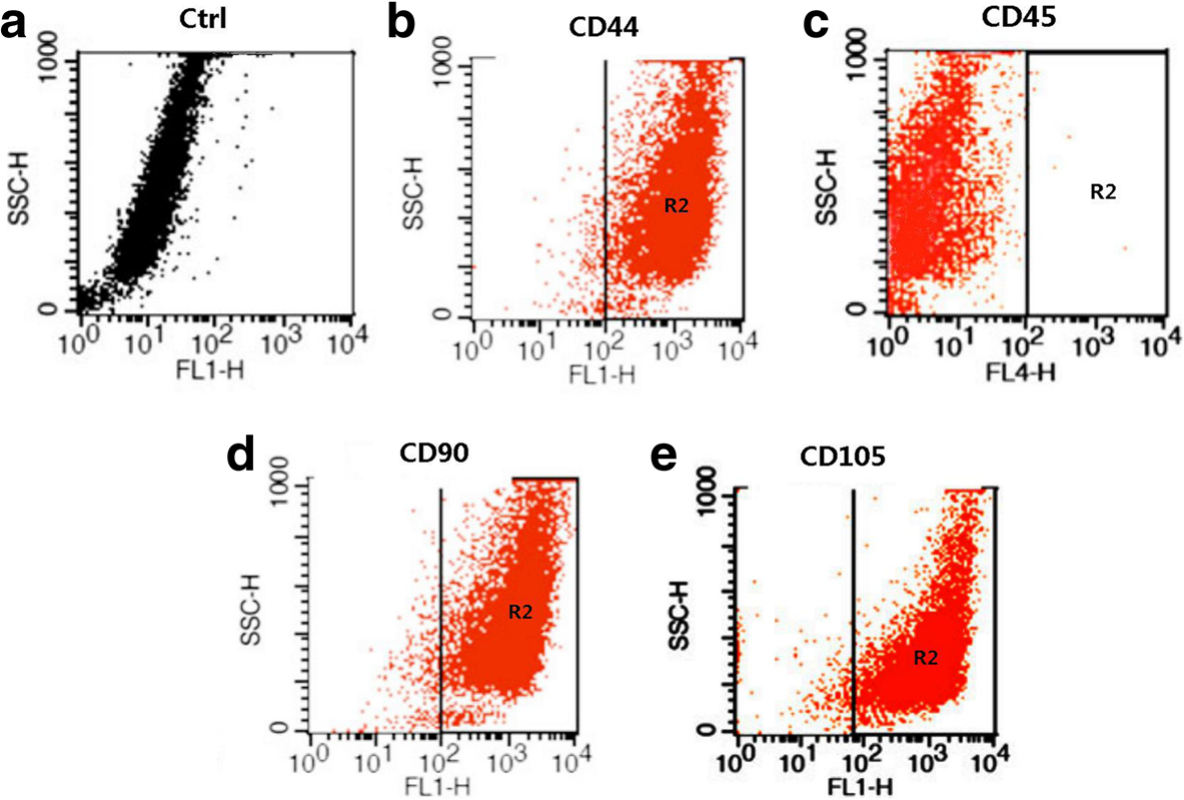
Flow cytometry analysis of ADSCs from adipose tissue of Wistar rats. The cells were analysed and presented with high expression of CD44 (98.1%), CD90 (98.7%) and CD105 (99.2%) (**b**, **d** and **e**) and the lack of expression of CD45 surface antigen (0.1%) (**c**) compared with the control (**a**)

The cells began to adhere within 72 h after inoculation, and at first, the cells grew slowly, unevenly, and presented in various forms, such as spindle and oval. After changing the medium two or three times, the nonadherent cells were removed. The cells grew faster after 2 weeks and appeared to be uniform long polygons, while the whole looked similar to a spiral. Flow cytometry dot plots represented quantification of markers for ADSCs. The cells were analysed and presented the typical features of ADSCs, with high expression of CD44 (98.1%), CD90 (98.7%) and CD105 (99.2%) (Fig. 1b, d and e) and the lack of expression of CD45 surface antigen (0.1%) (Fig. 1c). This suggests that these cells had typical surface markers of mesenchymal stem cells without the surface marker of blood cells, which should be highly pure ADSCs.

### Osteogenic test of ADSCs

**Fig. 2:**
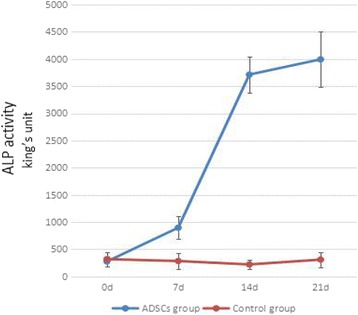
ALP activity of ADSCs lysis solution after osteogenesis induction (king’s unit, *n* = 6). ALP activity of the ADSCs group showed a gradual increase with the extension of induction time. The control group was set by the rat fibroblasts, and ALP activity of their lysis solution did not increase. *ADSCs* adipose-derived stem cell

The ALP activity of the ADSCs increased gradually when the ADSCs group was osteogenesis induced for 7 d, and the ALP activity of the ADSCs group was significantly higher than the control group (*P* < 0.05), which had the equivalent of rat fibroblasts osteogenic induced. The ADSCs group’s ALP activity was improved more when induced 14 d and 21 d (*P* < 0.01). As the results show, ALP activity showed a gradual increase with the extension of induction time. In contrast, the control group’s ALP activity did not increase (Fig. 2, Table 1). This suggests that ADSCs could form osteoblasts under certain conditions but fibroblasts could not.

**Table 1:** ALP activity of ADSCs lysis solution after osteogenesis induction (x ± s, n = 6)

Group	ALP activity (king’s unit)			
	0 d	7 d	14 d	21 d
ADSCs group	275.5 ± 93.5	896.0 ± 208.8*	3714.7 ± 331.6**	3994.7 ± 506.9**
Control group	317.2 ± 133.2	283.2 ± 150.4	220.2 ± 91.5	308.8 ± 139.2

Compared to the control group: *P < 0.05, **P < 0.01

*ADSCs* adipose-derived stem cells, *ALP* alkaline phosphatase

### Adhesion rate and survival rate of ADSCs on HDB

Adhesion efficiency of ADSCs on HDB was estimated by digestion briefly by trypsin and count. The average adhesion rates of ADSCs osteogenesis group and ADSCs group were 74.7% and 73.9%. Even more surprising, the survival rates were nearly 100%. On the contrary, there were not much cells adhering to the bottom of the 96-well culture plate without HDB (Table 2). Presumably 4 h were not enough for ADSCs adherence on flat growth environment. HDB’s cavernous space was obviously more suitable for cells to adhere within a short time and survive. However in 4 h, ADSCs-HDB adhesion was not very tight, and trypsin could quickly digest the cells.

**Table 2:** Adhesion rate and survival rate of ADSCs on HDB (x ± s, *n* = 10)

Group	Adhesion number (*10^3^)	Adhesion rate (%)	Survival number(*10^3^)	Survival rate (%)
ADSCs osteogenesis group	74.7 ± 5.71	74.7 ± 5.71*	72.5 ± 4.60	97.1 ± 1.90*
ADSCs group	73.9 ± 5.97	73.9 ± 5.97*	71.7 ± 5.44	96.9 ± 0.84*
control group	14.1 ± 9.22	14.1 ± 9.22	10.4 ± 6.50	55.8 ± 11.50

Compared to the control group: *P < 0.01

*ADSCs* adipose-derived stem cells, *HDB* heterogeneous deproteinized bone

### Gross observation

**Fig. 3:**
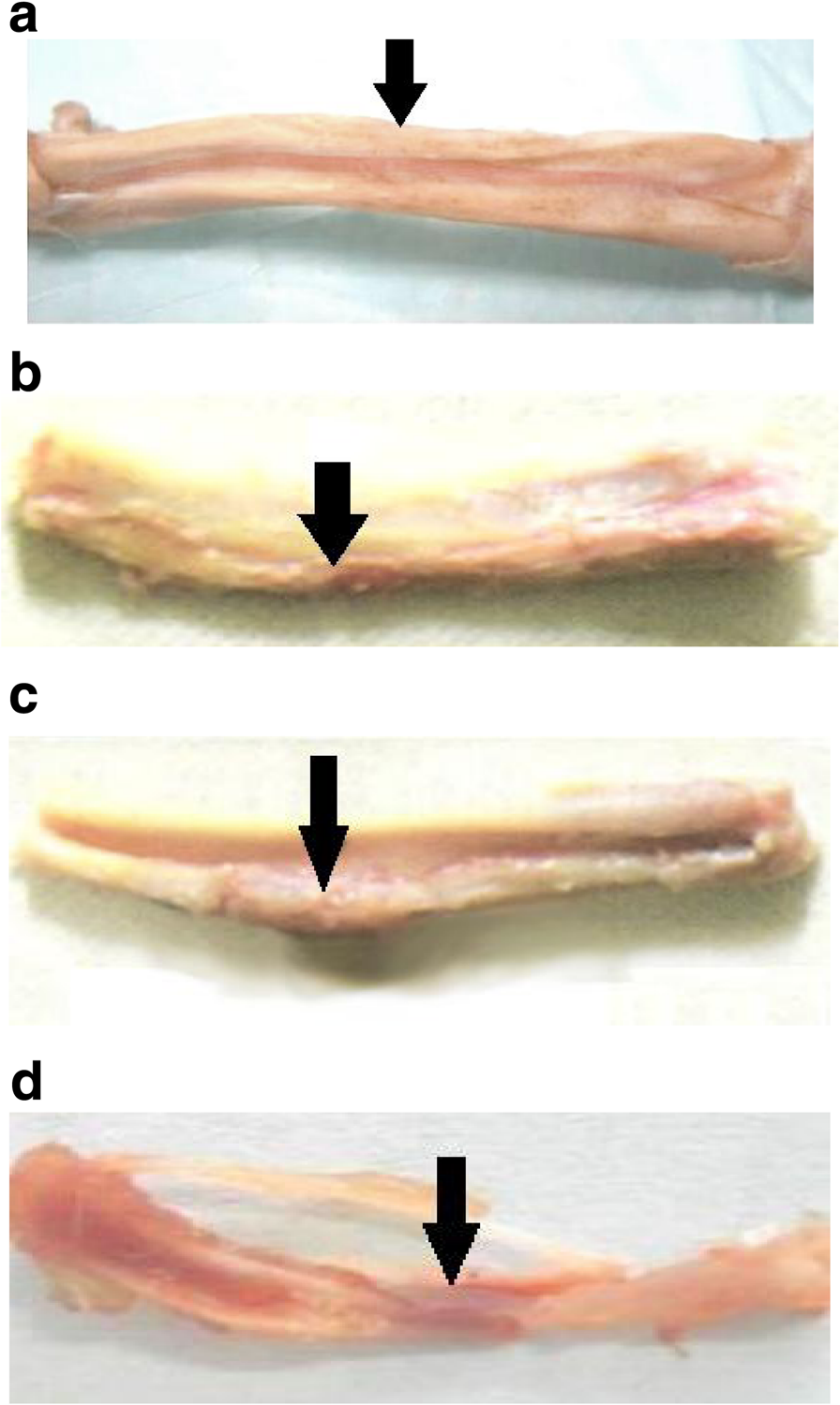
Gross observation of repair of bone defects at 8 weeks. **a** In ADSCs osteogenesis group, the appearance of the defect was consistent with the unmarred bone. **b** In ADSCs group, the bone defect of was fully filled with a large number of newborn bone tissue, but the implant was clearly identifiable. **c** In the negative control group, the line was clear with bleak HDB and the joints were slightly thicker than the upper or lower bone. **d** In blank control group, there was kermesinus tissue between the two ends

In ADSCs osteogenesis group, the average total number of cells implanted was 7.47 × 10^5^ cells with an average cell viability of 97.1%. In ADSCs group, the average total number of ADSCs was 7.39 × 10^5^ cells with an average viability of 96.9%. In the negative control group, no living cells were on HDB. To determine whether the three implant materials yielded different effects in vivo, osteogenesis experiments on rat models were performed. As shown in Fig. 3, the healing process of each group is clearly displayed. Two weeks after surgery in the ADSCs osteogenesis group, the new callus scab was seen at both ends of the defect area, which obviously formed the bridge between the defect and the implant. The bone defect area was completely filled with newborn bone tissue or the osteogenesis-ADSCs-HDB complex after 4 weeks. The appearance of the defect in this group at 8 weeks after operation was consistent with the unmarred bone, which was yellowish and difficult to recognize (Fig. 3a). In the ADSCs group, at the end of 4 weeks after surgery, an obvious new callus scab formed the bridge of the defects with the ADSCs-HDB composites. The bone defect of 8 weeks after surgery was fully filled with a large number of newborn bone tissue, and the connection was strong, but the implant was clearly identifiable, and the colour was yellow (Fig. 3b). In the negative control group, only a small amount of callus formation was visible in the bone defect 2 weeks post-operation. There was bone sclerosis in the two severed ends in 8 weeks, and the line was clear with bleak HDB. The joints were slightly thicker than the upper or lower bone, but the connection was not strong (Fig. 3c). In the non-transplanted blank control group, there was kermesinus tissue between the two ends and osteogenesis of the defect centre was not visible 4 weeks post-surgery. After 8 weeks, if the specimen was cut longitudinally, it was easy to find that the bone marrow cavities were closed completely and dark red connections were not true bone tissue (Fig. 3d).

### Molybdenum target X-ray examinations

**Fig. 4:**
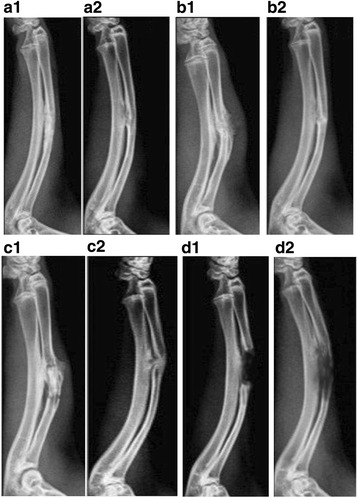
Radiographic examination of repair of bone defects. **A1, A2** In ADSCs osteogenesis group, the bones were well connected, a marrow cavity appeared, and the bone defect was almost impossible to detect. **B1, B2** In the ADSCs group, the bone marrow cavities became interconnected, but the defect was still obvious. **C1, C2** In the negative control group, fracture lines were clear and the marrow cavity did not appear. **D1, D2** In the blank control group, the bone defect was still visible. **A1, B1, C1, D1**: 4 weeks post-surgery; **A2, B2. C2, D2**: 8 weeks post-surgery

To compare which method yielded the best performance in vivo, the bone repair on the rat model was imaged by molybdenum target X-ray. The ADSCs osteogenesis group showed surprising improvement during the healing process; the density of the bone callus was increased significantly from 4 weeks to 8 weeks post-operation. Furthermore, the bones were well connected, a marrow cavity appeared, and the bone defect was almost impossible to detect (Fig. 4 A1, A2). In the ADSCs group, the cortical bones were being remodelled and the bone marrow cavities began to become interconnected at 8 weeks post-surgery, with the defect still obvious (Fig. 4 B1, B2). In the negative control group, fracture lines were clearly presented, the high-density bone calluses were connected with the broken end of the defect bone (Fig. 4 C1). Even in 8 weeks, the marrow cavity did not appear (Fig. 4 C2). In the blank control group, osseous bridges were not observed 4 weeks post-surgery (Fig. 4 D1). Very low-density callus analogues were observed, whereas the bone defect was still visible at 8 weeks post-surgery (Fig. 4 D2).

The bone formation scores were then further evaluated according to the density value of the defect. The statistical data showed that the density values were similar between the ADSCs osteogenesis group and the ADSCs group. But they were significantly higher than the density value of the blank control group 4 weeks post-surgery, which revealed that HDB combined with ADSCs improved the osteogenesis process. Interestingly, the bone density of the ADSCs osteogenesis group and the ADSCs group gradually decreased in 4 to 8 weeks, suggesting that the two groups began bone reconstruction. The negative control group obviously was still in the repair instead of the reconstruction process, and the bone density increased slightly. The blank control group also naturally formed a small amount of bone, but could not really repair the segmental defect (Table 3).

**Table 3:** Callus density values in rats at different times after defect operation (x ± s)

	Group	4 w (N = 10)	8 w (*N* = 5)
A	ADSCs osteogenesis group	0.403 ± 0.010*	0.292 ± 0.022*
B	ADSCs group	0.391 ± 0.015*	0.341 ± 0.030*
C	Negative control group	0.334 ± 0.021*	0.372 ± 0.019*
D	Blank control group	0.019 ± 0.012	0.088 ± 0.026

Compared to the control group: *P < 0.01

*ADSCs* adipose-derived stem cells

### Histological features

**Fig. 5:**
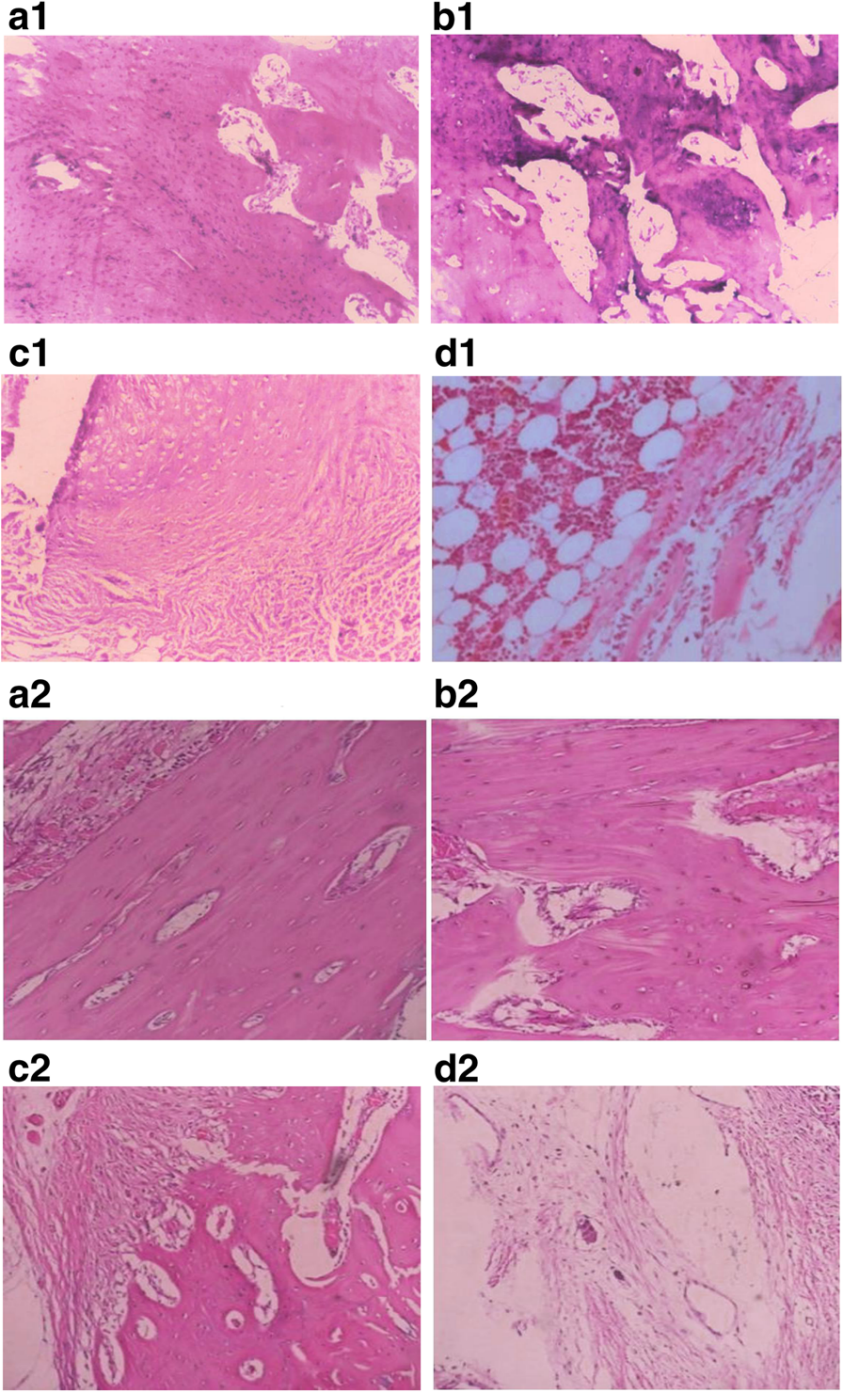
Histological image of bone repair. **A1, B1** An osteoid component appeared between the bone defects and the ADSCs-HDB composites of the ADSCs osteogenesis group and ADSCs group. **C1** The joints at the defect sites of the negative control group were fibrous tissue. **D1** In the blank control group, the inflammation was alleviated, all kinds of cells were mixed, and the structure was disordered. **A2, B2** The lamellar bone structure and the bone marrow cavity appeared, and its vascular arrangement was uniform in the ADSCs osteogenesis group, irregular in the ADSCs group. **C2** In the negative control group, the joint was mainly fibrous. **D2** Inflammatory cells were sometimes visible in the fibrous tissue of the blank control group. **A1, B1, C1, D1**: 4 weeks post-surgery; **A2, B2. C2, D2**: 8 weeks post-surgery

The entire osteogenesis process was traced by histological examination. Histological analysis was used to detect inflammatory cell infiltration and the formation of trabecular. Inflammatory cells were observed in every group 2 weeks after surgery, although the non-transplanted blank control group had significantly more inflammation in the bone defect. At 4 weeks after surgery, an osteoid component appeared between the bone defects and the ADSCs-HDB composites of the ADSCs osteogenesis group and ADSCs group. There were a large number of vessels at the material edge, which were mainly from the surrounding soft tissue and bone marrow cavity (Fig. 5 A1, B1). The joints at the defect sites of the negative control group were fibrous tissue, and vessels were hardly observed (Fig. 5 C1). In the blank control group, the inflammation was alleviated, all kinds of cells were mixed, and the structure was disordered (Fig.5 D1). At 8 weeks post-surgery, in the ADSCs osteogenesis group, it was difficult to identify the implanted bone and the new bone tissue. The new bone was connected with the bone defect, which showed the continuity of bone. Moreover, the lamellar bone structure appeared, and the bone marrow cavity was observed. Its vascular arrangement was uniform and orderly, and the morphological structure was close to the shape of normal cortical bone (Fig. 5 A2). At the same time, the microscopy results obtained from the ADSCs group showed that woven bones gradually transformed into lamellar bones. The vascular network was still disorganized, the diameter of vessels was different and the distribution was irregular (Fig. 5 B2). In the negative control group, the joint to the decomposing HDB was mainly fibrous and showed few irregular blood vessels 8 weeks post-surgery (Fig. 5 C2). Comparatively, inflammatory cells were sometimes visible in the fibrous tissue in defect sites of the blank control group (Fig. 5 D2).

## Discussion

There are deficiencies in the methods of treating bone defects, and differentiable stem cell transplantation is a new option for repair. In this experiment, we used a conventional surgical procedure to obtain fat from the bilateral groin of the donor Wistar rats, and the obtained ADSCs were used in other Wistar rats to ensure the allogeneic nature of the stem cells. In the medical field, the therapeutic effect of allogeneic stem cells on fracture patients was more practical and meaningful. After being osteogenesis induced in vitro, allogeneic ADSCs were seeded into HDBs to form tissue-engineered bone. Then ADSCs-HDB composites were used to repair the central part of a radial bone defect and compared with the control group in 8 weeks.

The role of seed cells has been controversial. It was reported that the need for healing of a bone defect could be met by simply applying scaffold materials or compound growth factors with materials [9]. However, there was also the opposite view that materials could not repair the bone defect without seed cells. Some studies found that without the help of seed cells there was only a little bone formation on the edge of the bone fault, and the main fillers were the residual material [10]. It also hints that autologous cells could migrate to repair bone defects in the small defects by the conduction effect of the scaffold materials. However, in the process of a wide range of defect repairs, seed cells were essential.

Bone marrow MSCs are the superstars of seed cells because they are undifferentiated precursor cells. Their cell phenotype differentiation is not mature, and allotransplantation rejection of is weak [11]. When amplificated bone marrow MSCs of different concentrations were injected intravenously into the volunteers, immune rejection was not found even when the input volume reached 5 × 10^7^, which suggests that these cells were well tolerated by the body [12]. In addition, the source of bone marrow MSCs is wide and easy to collect. Therefore, many researchers in the early stage used local bone marrow injection to treat bone defects [13]. However, further studies showed that on one hand, the limited number of bone marrow MSCs could not guarantee an effective concentration, and on the other hand, it was difficult to stimulate osteoblast differentiation due to its multidifferentiation status. Exciting research confirmed that after short-term cultivation, the number of MSCs could amplify hundreds of millions of times without losing their osteogenic ability, which allowed them to differentiate directly into osteoblasts, to meet the needs of the large bone defect repair [14]. It was found that MSCs not only provided the osteogenic cells necessary for bone formation but also secreted growth factors that promoted the repair of defects [15]. It is not very clear whether ADSCs provide the same function in repairing bone defects. It was the original intention of this experiment to elucidate the repair mechanism of ADSCs and the long-term transformation in vivo. And a study of the growth factors secreted by ADSCs, which are labelled with green fluorescent protein (GFP), is underway.

Studies on the construction of tissue-engineered bone by ADSCs have mainly been carried out using endogenous cells or in vivo in nude mice [16, 17]. There were reports that ADSCs were similar to BMSCs, but they had weak immunogenicity [18, 19]. However, there are not many reports on the repair of bone defects using allogeneic ADSCs. In this experiment, allogeneic ADSCs were utilized to construct tissue-engineered bone to repair segmental bone defects in the radii of Wistar rats. There were inflammatory responses, such as redness and seepage 1 week after the implanted operation, but wounds healed well. In 8 weeks, necrosis, fester, and effusion were not seen from the surrounding tissue of the specimen material, and the composites combined with the surrounding soft tissue naturally without envelope formation. H&E-stained samples were not infiltrated by inflammatory cells, suggesting the allogeneic ADSCs and HDBs were compatible with receptors and with a mild immune rejection. This finding will lay the foundation for ADSCs as candidates to provide the seed cell library for bone tissue engineering.

The implanted cells are easily lost without cell carriers, so the effective cell concentration is hard to maintain at the implant site, and their osteogenic capacity is limited [20]. Suitable extracellular scaffold materials can provide the cells with a site for adhesion, growth and reproduction space, which is conducive to tissue regeneration [21]. Many studies have shown that osteogenesis of carriers composited with MSCs was significantly superior to that of seed cells without scaffold. The scaffold materials of bone tissue engineering should not only have good porosity and mechanical strength but also be beneficial for cell adhesion, proliferation, matrix deposition and angiogenesis [22]. At present, scaffold materials is an active research area and focused mainly on biological ceramics and degradable polymer organic materials [23–25]. Porous ceramic scaffolds covered with MSC were implanted in the rat’s defect, which obtained a better repair effect compared with pure ceramic stents [26]. Due to the diversity of bone defects and shortcomings of synthetic scaffold material, such as biocompatibility, mechanical properties, researchers are still looking for the ideal bone tissue engineering scaffold material.

Bone-derived materials are another trend in the study of scaffold materials, which are mainly demineralized bone and deproteinized bone [27, 28]. Both have good biocompatibility and osteogenesis effects and can be used for bone and cartilage tissue engineering scaffold material. They are rich sources with easy production and low cost. Superior to synthetic materials, they provide a convenient, fast, and cheap approach for the biomimetic transplantation of bone and joint. Studies on the successful construction of tissue-engineered bone using demineralized bone have been reported [29–31]. In addition, the deproteinized bone also has a natural reticular pore system. The characteristics of the pore traffic, pore size and components from mammals are similar. So they are more in line with the physiological requirements of bone repair [32]. Without protein as an antigen, it is difficult to induce an immune response, even for the heterogeneous bone. Research has shown that, comparatively speaking, the mechanical strength of the deproteinized bone was significantly stronger than the demineralized bone, so that its pore system might not be compressed. After implantation, the demineralized bone could not maintain the mesh pore system completely under the pressure of the swelling tissue, which affected the proliferation of cells within. Then this affected the final osteogenesis [32]. In contrast, the deproteinized bone, mainly composed of hydroxyapatite, had good biodegradability. And its degradation can release many calcium phosphate ions, the deposition of which is helpful to promote calcification and osteogenesis [33]. Therefore, deproteinated bone was selected as a scaffold in this experiment.

To explore the feasibility of HDB as a scaffold material and ADSCs as the seed cells, adipose MSCs were amplified in vitro, seeded in the bone-derived material, and then the composite was implanted in the bone defect. Its effect on bone repair was assessed in the body. Experimental results showed that in the experimental group (ADSCs osteogenesis group and ADSCs group), after the ADSCs transplantation, the newly formed bone was evident with time. Without periosteum, the osteogenesis is osteochondral bone. In the first 2 to 4 weeks after the implantation of ADSC seeded scaffolds, the cartilage analogue was formed first. As the time extended, some cartilage analogue gradually became ossified. In 8 weeks, some mature woven bone and a small amount of bone marrow cavity formed.

The gross observation, Molybdenum target X-ray examinations and histological measures showed that the defects were filled with new bone completely if the ADSCs-HDB implanted. Compared with the ADSCs group, the marrow cavity of the ADSCs osteogenesis group was remodeled earlier and the new blood vessels were more regular.

It is also proved that after ADSCs were osteoinducted, the repair of bone defect was better. ADSCs and HDB were good options for repairing bone defects. Without a doubt, the natural three-dimensional mesh pore system of the bone-derived material provides enough space for cultivation, growth and secretion of extracellular matrix for seed cells. In addition, its chemical composition has good cell and tissue compatibility, which made the acceptor’s vascular system grow into the material and provide a better microenvironment for the exchange of gases and nutrients of the osteoblasts and surrounding tissue. In the negative control group, the reason of mild osteogenesis at the junction might be some active ingredients in bone-derived materials, such as residual bone morphogenetic protein (BMP). These could induce osteogenesis of autologous mesenchymal stem cells in the bone.

The anode of the Molybdenum target X-ray is made of molybdenum (Mo), while the anode of an ordinary X-ray tube is made of tungsten (W). Molybdenum target X-ray is soft because the tube voltage is generally 30 kV, 20~25 kV less than the tungsten target [34]. This feature determines that its wavelength is 0.63 or 0.71, which is 3.0–4.0 times longer than tungsten target. Its X-ray is easily absorbed by soft tissue and small bone and suitable for the small bodies and thin tissue of some animals such as the rat. According to its characteristics, the density difference between organs is increased and X-ray image contrast is improved since computer X-ray photography is combined. A better image effect in special tissues using molybdenum target X-ray can be obtained compared with the tungsten target X-ray. This can improve the accuracy of X-ray diagnosis. Therefore, it is one of the most common and effective methods for breast examination and disease diagnosis [35]. Computer X-ray imaging scanning technology can compensate for the shortcoming of conventional X-rays in small sizes and for the tiny bones of the rat. In general animal experiments, biomechanical methods are usually used to measure the healing of bone scabs. However, this method to measure bone strength can only be used in the later stage, and it cannot show the whole process from early to late. Therefore, it has a great limitation. By using the histogram principle of computer image analysis, combined with molybdenum target X-ray, the callus formation was measured in early and late stages of the calcification process, which objectively reflected the bone strength changes in the process of fracture healing.

Long-term effects of allogeneic ADSCs engineered bone for repairing tubular bone defects and mechanisms of immune tolerance in vivo are questions for further exploration.

## Conclusions

In this experiment, the composite of allogeneic ADSCs and HDB had a strong osteogenic ability and was used to repair rat segmental bone defects successfully. This composite is promising to serve as grafting material in bone tissue engineering.

## Abbreviations

ADSCs: Adipose-derived stem cells
ALP: Alkaline phosphatase
AN: adhesion number
DMEM: Dulbecco’s modified Eagle’s medium
HDB: Heterogeneous deproteinized bone
MSCs: mesenchymal stem cells
PBS: Phosphate-buffered saline
